# Effects of Ni doping on various properties of NbH phases: A first-principles investigation

**DOI:** 10.1038/s41598-017-06658-2

**Published:** 2017-07-26

**Authors:** Yang Wu, Zhongmin Wang, Dianhui Wang, Zhenzhen Wan, Yan Zhong, Chaohao Hu, Huaiying Zhou

**Affiliations:** 1 School of Materials Science and Engineering, Guilin University of Electronic Technology, Guilin, 541004 P.R. China; 2 Guangxi Key Laboratory of Information Materials, Guilin University of Electronic Technology, Guilin, 541004 P.R. China; 3 Guangxi Experiment Center of Information Science, Guilin, 541004 P.R. China

## Abstract

Changes in the stability, hydrogen diffusion, and mechanical properties of the NbH phases from Ni-doping was studied by using first-principles methods. The calculation results reveal that the single H atom adsorption is energetically favorable at the tetrahedral interstitial site (TIS) and octahedral interstitial site (OIS). The preferred path of H diffusion is TIS-to-TIS, followed by TIS-to-OIS in both Nb_16_H and Nb_15_NiH. Ni-doping in the Nb_15_NiH alloy lowers the energy barrier of H diffusion, enhances the H-diffusion coefficient (*D*) and mechanical properties of the Nb_16_H phase. The value of D increases with increasing temperature, and this trend due to Ni doping clearly becomes weaker at higher temperatures. At the typical operating temperature of 400 K, the D value of Nb_15_NiH (TIS) is about 1.90 × 10^−8^ m^2^/s, which is about 80 times higher than that of Nb_16_H (TIS) (2.15 × 10^−10^ m^2^/s). Our calculations indicated that Ni-doping can greatly improve the diffusion of H in Nb.

## Introduction

Membrane reactors, used for the separation and purification of dense hydrogen, are one of the most important components in industrial hydrogen production by the steam reforming of natural gas^1, 2^. Currently, although Pd and its alloys have been widely used for hydrogen separation and purification, their disadvantages such as high price and scarcity are also obvious. Over the last few decades, researchers have gradually shifted their attention to group VB transitional metals (V, Nb, and Ta) due to their potential of hydrogen permeability and relatively lower price^3^. Among them, niobium (Nb) has been well regarded as one of the most promising hydrogen separation materials, since Peterson *et al*. reported that it possesses excellent high-temperature mechanical properties as well as corrosion resistance^4–6^. Furthermore, Nb and its alloys also have been extensively used in hydrogen-related high-temperature structural applications, such as the diverter and nuclear material at the International Thermonuclear Experimental Reactor (ITER), due to their strong resistance to corrosion, high melting point, excellent mechanical properties, and small cross section of neutron absorption^4–8^. However, Nb alloys often have poor resistance to hydrogen embrittlement and therefore are limited in their practical applications^9–12^.

Further exploring Nb-based alloys with other elements is one solution to the above problem. Watanabe *et al*. revealed that the addition of W and Ru decreases the hydrogen solubility in Nb and therefore improves its resistance to hydrogen embrittlement^9, 11^. Hu *et al*. reported that the addition of W can improve the mechanical properties of the Nb_16_H phase, decrease the structural stability of the Nb_15_WH (TIS) phase, lower the diffusion barrier of H, and enhance diffusion paths for H^13, 14^. In addition, Ni is an effective catalytic component and widely used in metal-based alloy compounds for hydrogen storage. Doping with Ni can decrease the sensitivity to impurity gas on the surface, thereby reducing the pollution caused by impurities. To the best of our knowledge, however, the fundamental work of Nb alloying with Ni has not been reported in the literature. It is necessary to study the structural and diffusion properties of Ni in the NbH phase by theoretical methods. Such calculations will contribute to the in-depth study of new Ni-based hydrogen permeation materials.

In this paper, we employ highly accurate first-principles method to investigate the effects of Ni doping on the structural stability, electronic structure, mechanical property, and H-diffusion behavior of the Nb_16_H phase. To compare with the experimental composition of 5 at% of Ni in Nb_16_H^15–17^, we purposively selected the composition of Nb_16_H in this work, and one Ni atom is added to Nb_16_H. Our calculated results revealed that the addition of Ni can greatly improve the diffusion properties of H and the mechanical property of Nb. At the same time, our results also provide a theoretical basis for further work on Nb-Ni-based alloys.

## Results and Discussion

**Figure 1:**
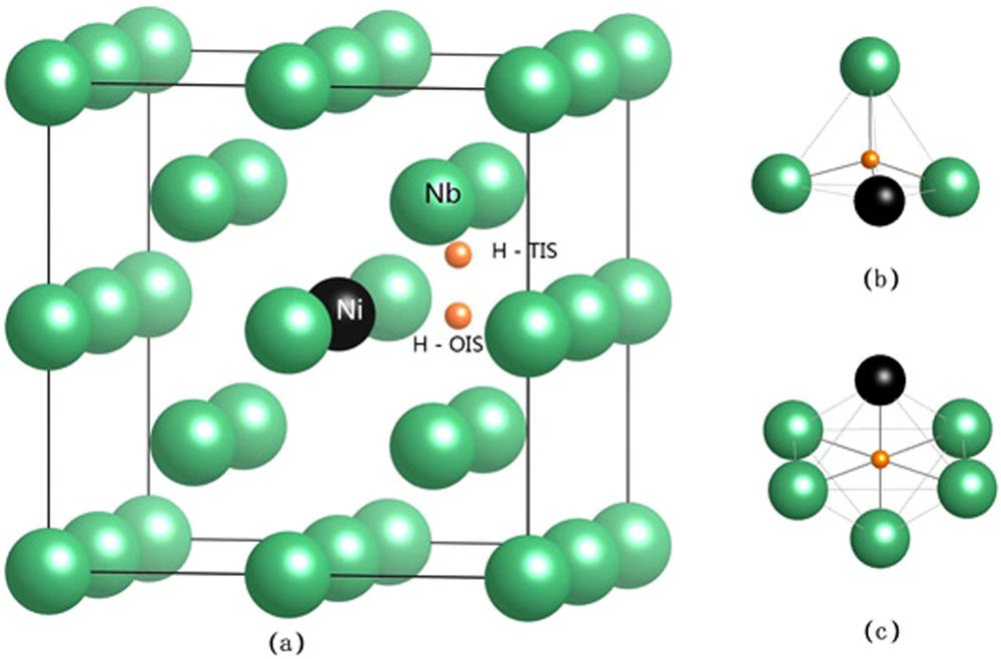
Schematic illustrations of (**a**) Nb_15_NiH, (**b**) tetrahedral interstitial site (TIS), and (**c**) octahedral site (OIS). The green and black spheres represent Nb and Ni atoms, respectively. The small orange spheres stand for various sites for the H atom.

In order to compare to the experimental results^15–17^, a 2 × 2 × 2 super cell containing 16 Nb atoms was built, and one of the Nb atoms was substituted by Ni. To study the diffusion behavior of H atom between the nearest sites, one H atom was placed at the tetrahedral interstitial site (TIS) and octahedral interstitial site (OIS) of Nb and Nb_15_Ni, respectively. In Nb_15_NiH (TIS), the Nb-H and Ni-H bond lengths are about 1.96 and 1.65 Å, respectively. After the doping of Ni atoms, the structure was changed from bcc to simple cubic due to the smaller atomic radius of Ni compared to Nb. However, the structure remained as the cubic type. As a typical example, Fig. 1(a) shows the schematic illustrations of Nb_15_NiH with H atom in TIS and OIS. For clarity, the corresponding atomic configurations of TIS and OIS are displayed in Fig. 1(b) and (c).

### Structural stability of Nb_16_H and Nb_15_NiH

To examine the effect of Ni-doping on the structural stability of Nb_16_H and Nb_15_NiH, the solution energy (*E*_s_) of interstitial H atom in Nb_16_H and Nb_15_NiH was investigated by means of the following formula:

1 $${E}_{S}=E{{\rm{Nb}}}_{{\rm{16}}-{\rm{x}}}{{\rm{Ni}}}_{{\rm{x}}}{\rm{H}}-E{{\rm{Nb}}}_{{\rm{16}}-{\rm{x}}}{{\rm{Ni}}}_{{\rm{x}}}-\frac{{\rm{1}}}{{\rm{2}}}E{{\rm{H}}}_{{\rm{2}}}$$

where $E{{\rm{Nb}}}_{{\rm{16}}-{\rm{x}}}{{\rm{Ni}}}_{{\rm{x}}}{\rm{H}}$, $E{{\rm{Nb}}}_{{\rm{16}}-{\rm{x}}}{{\rm{Ni}}}_{{\rm{x}}}$ and $E{{\rm{H}}}_{{\rm{2}}}$ are the total energies obtained from first principles calculations. The calculated *E*_s_ values of H-TIS and H-OIS in Nb and Nb_15_Ni are summarized in Table 1. The TIS and OIS models of Nb_16_H and Nb_15_NiH are all thermodynamically stable with negative solution energy. Moreover, the *E*_s_ of Nb_16_H (TIS) is obviously lower than that of Nb_16_H (OIS), which indicates that TIS is more energetically favorable than OIS for H in the *bcc* Nb. However, it must be pointed out that the *E*_s_ of TIS and OIS models in Nb_15_NiH alloys are very close to each other. It suggests that Ni-doping will increase the number of stable positions for H atom in the Nb_16_H phase.

**Table 1:** Calculated solution energy (*E*_*S*_) of interstitial H atom in Nb_16_H and Nb_15_NiH.

Sample	Nb_16_H	Nb_15_NiH
TIS	−0.43 eV	−0.389 eV
OIS	−0.14 eV	−0.384 eV

### Energy barrier of H diffusion in Nb_16_H and Nb_15_NiH

**Figure 2:**
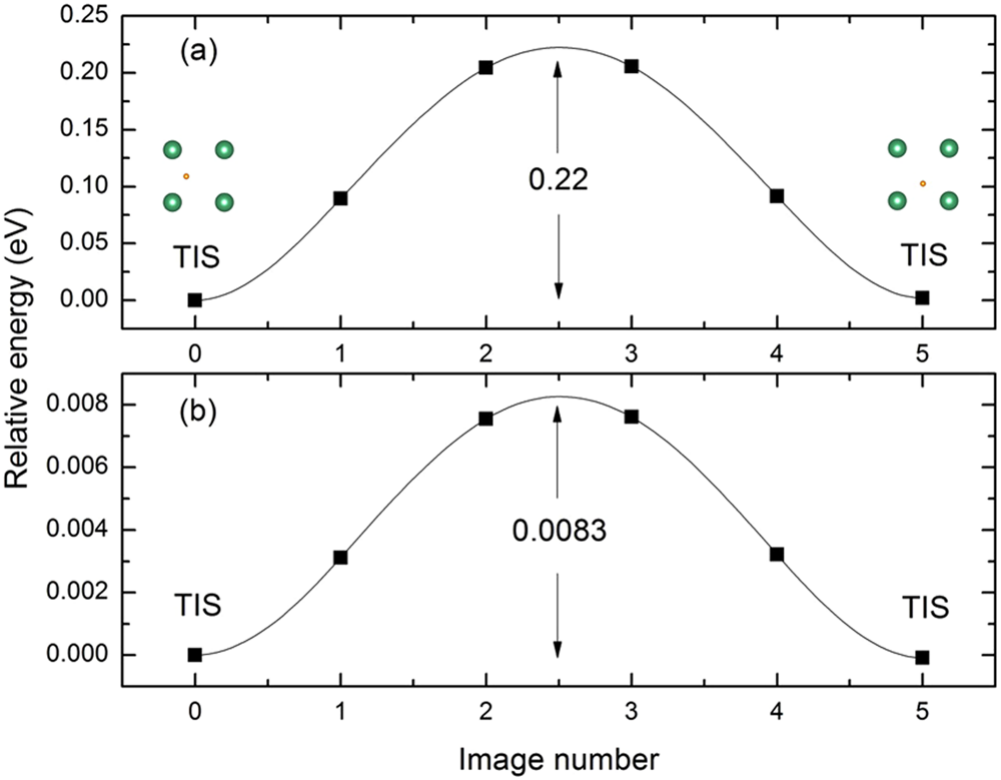
Energy barriers of H diffusion from TIS to TIS in (**a**) Nb and (**b**) Nb_15_Ni.

**Figure 3:**
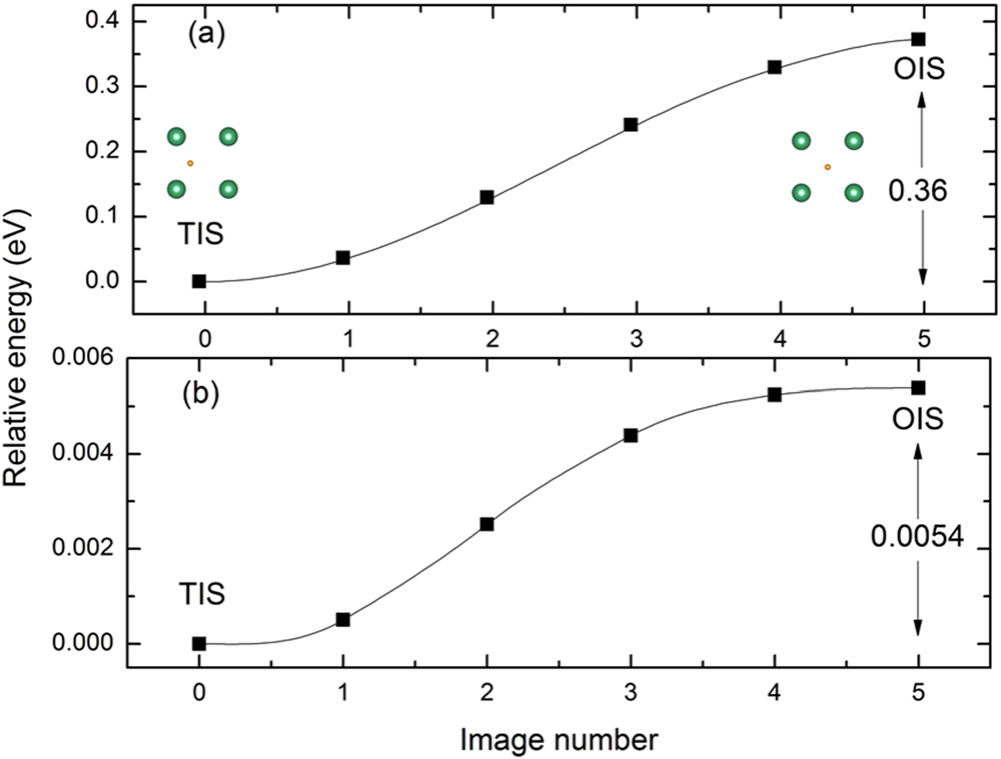
Energy barriers of H diffusion from TIS to OIS in (**a**) Nb and (**b**) Nb_15_Ni.

To investigate the effect of Ni-doping on H diffusion behavior, the climbing image nudged elastic band method (CI-NEB) is used to find out the minimum energy path and energy barrier for the H diffusion in Nb_16_H and Nb_15_NiH^18–21^. As shown in Figs 2 and 3, the possible paths of H diffusion in both samples are TIS → TIS and TIS → OIS. The energy barrier from TIS to TIS in Nb is 0.22 eV, which is lower than the corresponding value of 0.36 eV (at the saddle point) from TIS to OIS, suggesting that the diffusion path of H in bulk Nb should be mainly TIS → TIS rather than TIS → OIS. However, for H diffusion in Nb_15_Ni, a different trend is observed: the energy barriers of TIS → TIS and TIS → OIS are very close (0.0083 and 0.0054 eV, respectively). This indicates the preferred paths of H diffusion increase from single TIS → TIS to dual TIS → TIS and TIS → OIS. Namely, the additional diffusion paths and lower energy barrier will fundamentally lead to a higher H diffusion coefficient in Nb_15_NiH alloy. Therefore, the Ni-doping should have an important effect on the diffusion behavior of H in Nb.

### Diffusion properties of H in Nb_16_H and Nb_15_NiH

**Figure 4:**
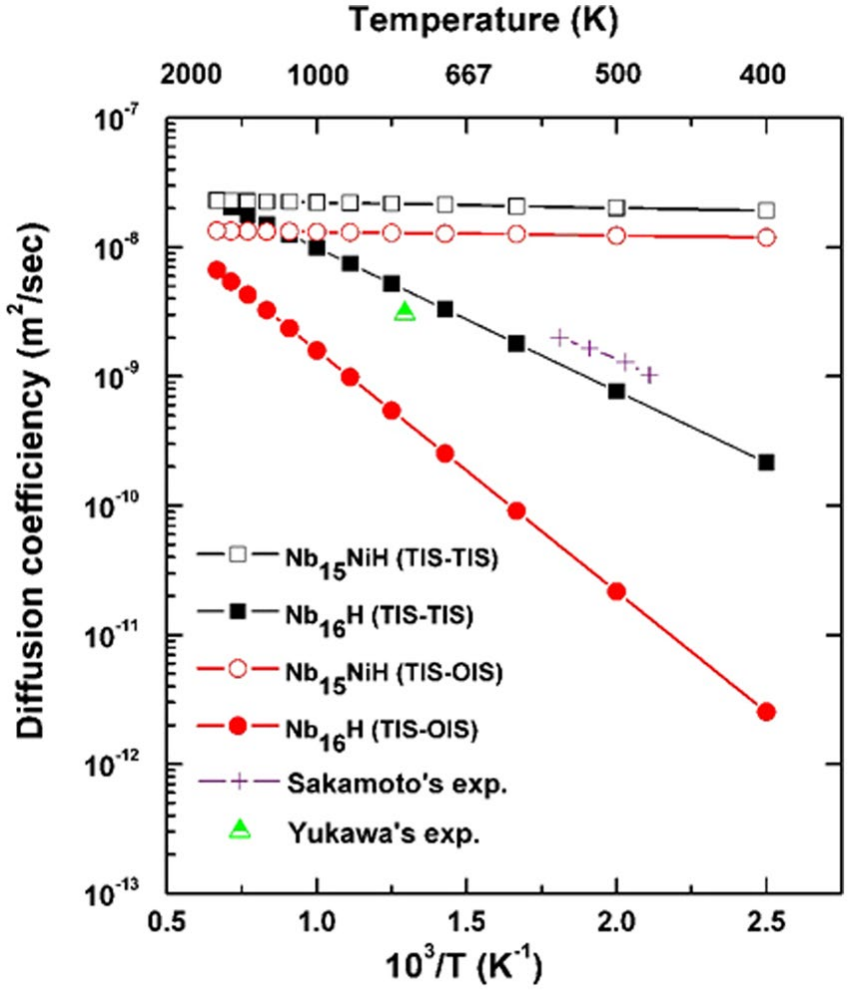
Diffusion coefficients of H along the various paths, as a function of reciprocal temperature. Experimental data are from the work reported by Sakamoto^25^ and Yukawa^26^.

The diffusion coefficient (*D*) is an important parameter for determining the diffusion velocity of H in Nb and Nb_15_Ni, providing quantitative information about the H diffusion^22, 23^. According to the Arrhenius diffusion equation, *D* can be expressed by

2 $$D={D}_{0}\,\exp (-Ea/kT)$$

where *D*_0_, *E*_a_, *k*, and *T* are the pre-exponential factor, diffusion energy barrier, Boltzmann constant, and absolute temperature, respectively. For a cubic metal, *D*_*0*_ can be expressed as

3 $${D}_{0}=\frac{1}{6}{r}^{2}{\rm{\nu }}$$

where *r* and *ν* are the jump distance and vibration frequency, respectively. We can calculate the vibration frequency *ν* according to Zener and Wert’s theory^24^, which is approximately expressed by

4 $${\rm{\nu }}=\sqrt{{\mathrm{2Ea}/\mathrm{mr}}^{{\rm{2}}}}$$

where *m* is the mass of the impurity atom. The mass of the H atom is already known (1.67 × 10^−27^ kg), the jumping distance of the TIS H in Nb and Nb_15_Ni is $a/2\sqrt{2}$, and that of the OIS H in Nb and Nb_15_Ni is *a*/4. Figure 4 shows the diffusion coefficient of H in Nb and Nb_15_Ni as a function of reciprocal temperature. The two phases exhibit different hydrogen diffusion behaviors depending on the operating temperature. In the case of Nb_16_H, the value of *D* clearly increases with increasing temperature. Meanwhile, our calculation results are consistent with the experimental data reported by Sakamoto and Yukawa^25, 26^. Note that the diffusion coefficient is greatly increased with the Ni doping, especially at low temperatures. At 400 K, the calculated *D* of Nb_16_H (TIS-TIS), Nb_15_NiH (TIS-TIS), Nb_16_H (TIS-OIS), and Nb_15_NiH (TIS-OIS) are 2.14 × 10^−10^, 1.90 × 10^−8^, 3.40 × 10^−12^, and 1.25 × 10^−8^ m^2^/s, respectively. Among the four paths, Nb_15_NiH (TIS-TIS) has the highest *D* value, which is about 80 times larger than that of the next one, namely Nb_16_H (TIS-TIS), followed by Nb_15_NiH (TIS-OIS). Moreover, for the TIS-TIS case, the effect from Ni-doping on *D* becomes weaker with increasing temperature. While in the TIS-OIS case, the *D* value of Nb_15_NiH remains higher in the full range of 400–1500 K.

### Mechanical properties of Nb_16_H and Nb_15_NiH

Now we examine the effect of Ni-doping on the mechanical properties of Nb hydride. The elastic constants of Nb_16_H (TIS and OIS) and Nb_15_NiH (TIS and OIS) are calculated for comparison. This value could be obtained by analyzing the difference in total energy between the original cell and deformed cell under a series of small strains. The obtained elastic constants are then used to calculate the bulk modulus (*B*) and shear modulus (*G*) from the Voigt-Reuss-Hill approximations^28, 29^. The Young’s modulus (*E*) is determined by means of *E* = *9BG*/(*3B* + *G*)^29^. After a series of calculations, the lattice constants (*a*), three independent elastic constants (*C*_*11*_, *C*_*12*_, and *C*_*44*_), and elastic moduli (*B*, *G*, and E) of various Nb_16_H and Nb_15_NiH phases are obtained (Table 2). The calculated values of pure Nb are also listed and compared with experimental results. The consistency between the calculated and experimental values proved the reliability of our calculation method.

**Table 2:** Calculated lattice constants (*a*), elastic constants (*C*_*11*_, *C*_*12*_, *C*_*44*_), bulk modulus (*B*), shear modulus (*G*), Young’s modulus (*Y*), and *B*/*G* of Nb_16_H and Nb_15_NiH phases.

Sample	*a* (Å)	Elastic	constants	(GPa)	*B* (GPa)	*G* (GPa)	*E* (GPa)	*B*/*G*
		*C*_*11*_	*C*_*12*_	*C*_*44*_				
Nb	3.304	280.6	127.58	21.92	178.95	37.20	104.37	4.81
Nb (exp.)^27^	3.305	246.00	133.00	28.00	171.00	37.20	104.20	4.60
Nb_16_H (TIS)	3.314	299.58	118.66	23.16	181.57	41.52	115.74	4.37
Nb_16_H (OIS)	3.315	286.25	120.79	12.83	176.34	30.08	85.39	5.86
Nb_15_NiH (TIS)	3.286	279.81	133.85	49.62	193.56	57.48	156.91	3.37
Nb_15_NiH (OIS)	3.285	276.81	129.08	51.34	181.19	59.26	160.30	3.06

The calculated results of pure Nb and experimental values^27^ are also included for comparison.

(i) We use the following criteria for mechanical stability: ${C}_{44} > 0,\,\frac{({C}_{11}-{C}_{12})}{2} > 0$, $B=\frac{({C}_{11}+2{C}_{12})}{3} > 0,$ ${C}_{12} < B < {C}_{11}$ ^30^. Table 2 presents the computed values of *C*_*11*_, *C*_*12*_, and *C*_*44*_ for the materials, showing that they are all mechanically stable. (ii) The shear modulus G represents the resistance to plastic deformation, while the bulk modulus B represents the resistance to fracture^31^. The Young’s modulus E can characterize the stiffness of a material, with a higher value in the stiffer material. The values of *B*, *G*, and E of Nb_15_NiH (TIS) and Nb_15_NiH (OIS) alloys are obviously larger than those of Nb_16_H (TIS) and Nb_16_H (OIS) phases. (iii) The values of *B*, *G*, and E of Nb_15_NiH (TIS) and Nb_15_NiH (OIS) alloys are also obviously larger than those of Nb_16_H (TIS) and Nb_16_H (OIS) phases. According to the empirical criterion proposed by Pugh^32^, if the value of *B* is about 1.75 times larger than *G*, the material will be ductile, otherwise fragile. The calculated *B*/*G* values listed in Table 2 show that all phases are ductile. These results suggest that the Ni-doping could help to improve the mechanical properties of Nb_16_H phase, and enhance the resistance to hydrogen embrittlement.

### Electronic properties of Nb_16_H and Nb_15_NiH

**Figure 5:**
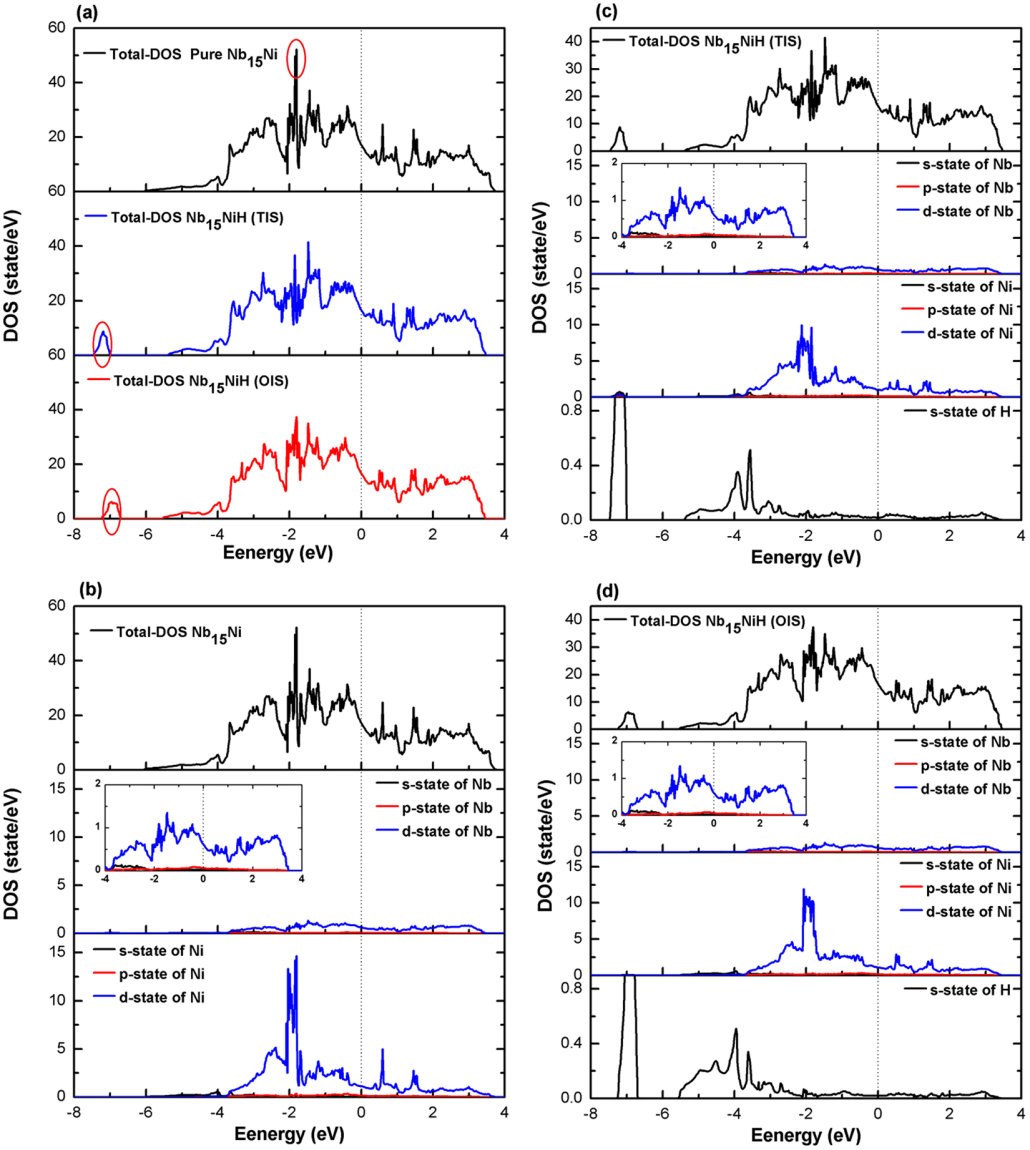
Total DOS of (**a**) Nb_15_Ni without and with H at the OIS and the TIS. The total and partial density of states of (**b**) Nb_15_Ni, (**c**) Nb_15_NiH (TIS), and (**d**) Nb_15_NiH (OIS). The Fermi level is set at zero.

The change of electronic properties in Nb_16_H and Nb_15_NiH due to Ni-doping was further investigated. Figure 5 shows the calculated total density of states (DOS) of pure Nb_15_Ni, Nb_15_NiH (TIS), and Nb_15_NiH (OIS). The highest DOS peak at about −2 eV is mainly contributed to the Ni-3d states, and it becomes lower with the addition of H. Similar results were obtained for hydrogen in vanadium by Luo and co-authors^33^. The partial density of states (PDOS) of Nb_15_NiH (TIS) and Nb_15_NiH (OIS) show obvious Ni-H and Nb-H hybridization interactions from the Ni-3d, Nb-4d, and H-1s states in the energy region from about −3.7 eV to the Fermi level (*E*_*F*_). In the cases of Nb_15_NiH (TIS) and Nb_15_NiH (OIS), the stronger DOS peaks of Ni-3d states at energies close to *E*_F_ imply that the Ni-H bond is stronger than Nb-H. These features of electronic structures signify that the Nb_15_NiH alloy should have stronger chemical bonding than Nb_16_H, which will result in improved structural stability and stronger mechanical properties.

**Figure 6:**
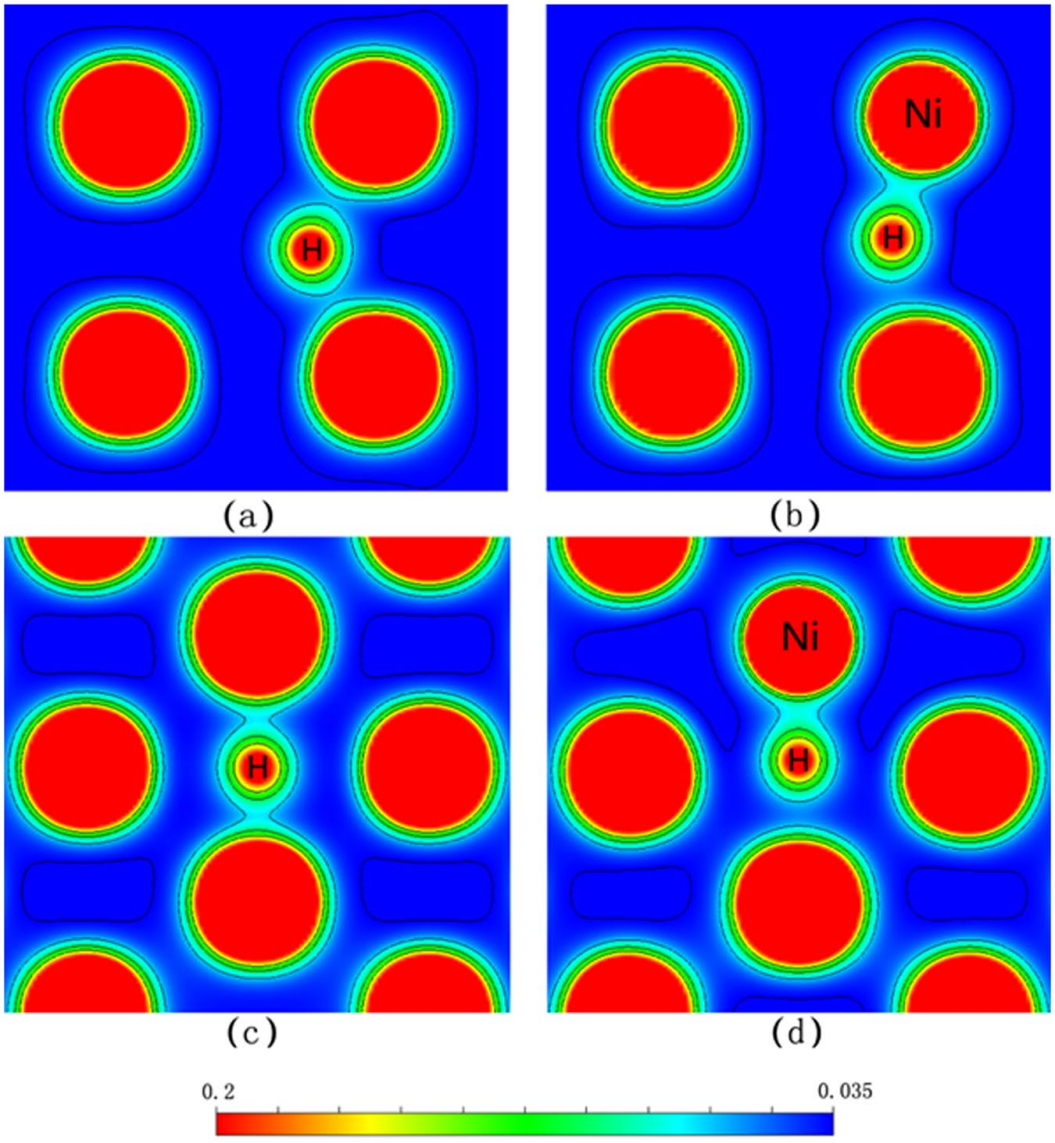
Charge density distribution of Nb_16_H and Nb_15_NiH with H at TIS site (**a**,**b**), H and at OIS site (**c**,**d**).

Figure 6 shows the charge density of Nb and Nb_15_Ni with H atom at the TIS and OIS sites, respectively. As shown in Fig. 6(a) and (c), the charge density distribution between H and Nb is symmetrical. However, from Fig. 6(b) and (d), the charge density between H and Ni is obviously higher than that between H and Nb after substituting Ni for Nb. This suggests the Ni-H bond is stronger than Nb-H bond, which is also in agreement with the above DOS analysis.

## Conclusions

We have investigated the structural stability, mechanical property, and hydrogen diffusion properties of H in pure Nb and Nb_15_Ni, using first-principles calculations in combination with empirical theory. The results show that Ni-doping can enhance the mechanical properties of Nb_16_H phase, decrease energy barrier of H diffusion, and improve H diffusivity in the Nb_16_H phase. The calculated density of states and charge density distribution reveal that the Ni-H chemical bond formed after Ni-doping is stronger than Nb-H in Nb_15_NiH, and this is directly responsible for the improved mechanical properties in Nb_15_NiH. The CI-NEB calculations indicate that the single H atom is energetically favorable for adsorption at the tetrahedral interstitial site (TIS) and octahedral interstitial site (OIS) in both Nb_16_H and Nb_15_NiH. The preferential path of H diffusion is from TIS-TIS, followed by TIS-OIS. The value of *D* increases, and the effect on *D* from Ni-doping clearly weakens at higher temperatures. At low temperatures, the value of *D* of Nb_15_NiH (TIS) is about 80 times larger than that of Nb_16_H (TIS) phase. The current results should help in future experimental investigations of the solubility, diffusion coefficients, and permeability of hydrogen in Nb hydrides.

### Computational methods

Our calculations were carried out using the well-known Vienna *ab initio* simulation package (VASP)^34, 35^, in the framework of density functional theory (DFT). The core-electron interactions were described by projected augmented wave (PAW) method^36, 37^. The exchange-correlations term was approximated by Perdew-Burke-Ernzerhof (PBE) corrected generalized gradient approximation (GGA) functions^38^. The electronic configurations 4d^4^s^1^ and 3d^8^4s^2^ were treated with the valences of Nb and Ni. The cutoff energy of plane wave was set to 360 eV, and the k-mesh of 5 × 5 × 5 was used in the Brillouin zone, which turns out to be sufficient to obtain convergence to less than 1.0 × 10^−6^ eV. Then, the atomic coordinates and crystal volume were relaxed with the conjugate gradient method, until the forces acting on all atoms are less than 0.01 eV/Å. These parameters ensured good convergence in the total energy. The migration barriers were calculated using the climbing image nudged elastic band method (CI-NEB)^18^. The calculation convergence and parameters stay the same for the ground state calculations.
